# Dysregulated eating behaviour and microbiota-based interventions targeting eating disorders and food addiction

**DOI:** 10.1080/19490976.2026.2647535

**Published:** 2026-03-29

**Authors:** Solveiga Samulėnaitė, Jan Rodriguez Parkitna, Daiva Baltriukienė, Elena Martín-García, Rafael Maldonado, Aurelijus Burokas

**Affiliations:** a Department of Biological Models, Institute of Biochemistry, Life Sciences Center, Vilnius University, Vilnius, Lithuania; b Institute of Biosciences, Life Sciences Center, Vilnius University, Vilnius, Lithuania; c Laboratory of Neuropharmacology, Department of Experimental and Health Sciences, Universitat Pompeu Fabra, Barcelona, Spain; d Department of Molecular Neuropharmacology, Maj Institute of Pharmacology of the Polish Academy of Sciences, Krakow, Poland; e Fundació Institut Hospital del Mar d'Investigacions Mèdiques (HMRIB), Barcelona, Spain; f Departament de Psicobiologia i Metodologia de les Ciències de la Salut, Universitat Autònoma de Barcelona (UAB), Cerdanyola del Vallès, Barcelona, Spain; g Université de Strasbourg (UNISTRA), INSERM UMR-S 1329, Strasbourg Translational Neuroscience and Psychiatry, Centre de Recherche en Biomédecine de Strasbourg, Strasbourg, France

**Keywords:** Eating disorders, binge eating, bulimia nervosa, food addiction, gut microbiota, microbiota–gut–brain axis, microbiota-based interventions, prebiotics, probiotics, postbiotics, FMT

## Abstract

The consumption of highly processed, hyperpalatable food in Western societies increases the risk of developing obesity and compulsive eating behaviors, which include food addiction (FA) and eating disorders (EDs), such as bulimia nervosa (BN) and binge eating disorder (BED). These behaviors can lead to a range of health consequences, including cardiovascular and metabolic diseases, cognitive impairments, and mental health disorders, among others. Given the evidence suggesting the involvement of the gut microbiota in regulating eating behavior, in recent years, scientists have sought to identify microbiota signatures associated with EDs and FAs. Multiple pro- and prebiotics, as well as other microbiota-based therapeutic interventions, have been suggested as preventive or treatment strategies for FA and EDs. To provide a comprehensive overview, this review is structured into two main sections. The first section describes compulsive eating behaviors, namely, BN, BED, and FA, recognizing their similarities and differences, and highlighting the importance of the proper distinction for the selection of targeted and effective treatment approaches. The second section provides an extensive summary of the recent research years behind the search for microbiota signatures and potential microbiota-based therapeutic interventions for managing EDs and FA in both humans and animal models.

## Introduction

According to the World Health Organization, over 16 million people worldwide have an eating disorder (ED) as of 2021, although the broader data from the Global Burden of Disease 2019 suggest that the prevalence might be much higher, reaching approximately 55.5 million individuals.^1^^,^^2^ The prevalence is alarmingly high among children and adolescents, affecting around 3.4 million individuals.^2^ In addition to age, sex also strongly influences the prevalence, with females being more prone to developing an ED.^3-5^ Currently, three EDs are officially acknowledged by the Diagnostic and Statistical Manual of Mental Disorders—5th edition (DSM-5), namely anorexia nervosa (AN), bulimia nervosa (BN), and binge eating disorder (BED), defining them as disturbances in eating behaviors accompanied by cognitive and psychosocial impairments and alterations in self-evaluation, commonly resulting in body weight changes.^6^ Another potential ED—food addiction (FA), which is not yet recognized by the DSM-5, has also drawn scientific interest due to its neurobiological and behavioral similarities to other EDs, and high prevalence, affecting 9-10% of the population.^7-10^ In agreement, a well-recognized tool for the diagnosis of FA, the Yale Food Addiction Scale version 2 (YFAS 2.0), is now widely used by clinicians involved in the management of ED.^11^

**Figure 1.**
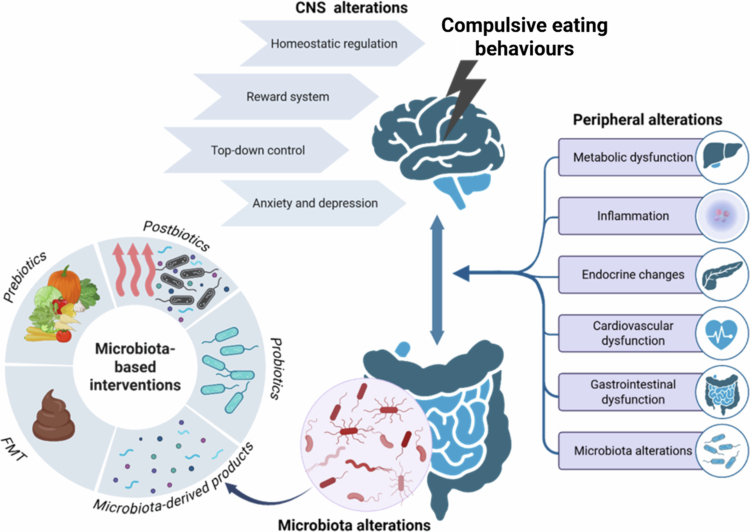
Central and peripheral alterations associated with EDs and microbiota-based strategies for their mitigation. Compulsive eating behaviors are accompanied by CNS and peripheral alterations, including changes in the gut microbiota composition. Modulation of the gut microbiota composition by prebiotics, probiotics, postbiotics, microbiota-derived products, and FMT could be used to potentially improve central and peripheral alterations. FMT—Fecal microbiota transplantation.

Both BED and FA are frequently associated with obesity due to an increased consumption of palatable food, while individuals with BN typically maintain stable body weight due to the compensatory behaviors.^5^^,^^12^ Regardless, given that all these EDs are linked to unhealthy eating habits, multiple organ systems are affected, leading to metabolic alterations, systemic inflammation, among others, and ultimately an increased risk of gastrointestinal and cardiovascular diseases as well as the development of type-2 diabetes. In addition to peripheral effects, the CNS is also affected, with one of the potential mechanisms being through inflammatory alterations. Systemic inflammation could ultimately lead to neuroinflammation affecting both homeostatic and reward-related food intake control centers and may also contribute to cognitive impairments and the development of other mental disorders, including depression and anxiety, among others.^13-18^ Remarkably, researchers have observed that the development of one ED increases vulnerability to the development of other EDs. Furthermore, EDs are closely connected with other mental disorders, such as depression, anxiety, obsessive-compulsive disorder, bipolar disorder, and even substance abuse.^5^ Indeed, the prevalence of depression and anxiety among people with EDs in the US was reported as 76.3% and 44.6%, respectively, for BN, and 65.5% and 59.0%, respectively, for BED, indicating a high rate of co-occurrence.^19^ Figure 1 summarizes the scope of this review, highlighting health consequences related to the development of compulsive eating behaviors, which include BN, BED, and FA, and microbiota-based strategies for their mitigation.

Numerous factors have been identified as contributing to vulnerability to EDs, including genetic predisposition and environmental components, such as family history, childhood trauma, and social environment, among others.^20^ However, the abundant availability of highly palatable foods in Western society represents another risk factor for ED development. Indeed, these foods are known to induce changes in the brain and facilitate the transition from controlled eating to compulsive overeating.^21^^,^^22^ In this review, we focus on EDs linked to palatable food overconsumption and obesity; therefore, AN is out of the scope of this review and will not be explored further.

In recent years, the gut microbiota has been extensively studied as a key modulator of health and disease, with multiple studies implicating it in the development of EDs.^23^ Existing data suggest that the microbiota alterations observed in EDs might not only be diet- and eating behavior-related consequences but also be a causal factor contributing to food choices and overall feeding behavior.^24^ Given the direct link between the gut microbiota alterations in EDs, this suggests that microbiota-based therapeutic interventions could potentially be a sufficient treatment strategy for these EDs. To date, most reviews predominantly focus on AN and obesity, with a low focus on BED, BN and especially FA; therefore, in this review, we provide an overview of the most recent scientific literature exploring the microbiota signature of BN, BED, and FA and summarize the microbiota-based therapeutic approaches that have been implemented so far to address these EDs in both humans and animal models.

## Dysregulated eating behavior

Both central and peripheral mechanisms are involved in maintaining the balance between energy intake and expenditure, thereby maintaining stable body weight. Within the CNS, the hypothalamus (HPT) is considered to be involved mainly in homeostatic regulation, while the mesolimbic system primarily regulates reward-related aspects of food intake; however, the functions of these two systems are interconnected.^25^^,^^26^ Under healthy conditions, the homeostatic system is modulated in response to physiological needs, whereas the activity of the reward system is modulated both by physiological needs and rewarding stimuli linked to palatable food and related cues.^27-29^ However, when the communication between peripheral hunger and satiety hormones, homeostatic, and reward systems is disrupted, it can result in a dysregulated eating behavior. Indeed, high consumption of palatable foods in vulnerable individuals not only dysregulates the homeostatic but also substantially alters the reward system. Eventually, the reward system overrides the homeostatic system, leading to increased cravings and motivation, which facilitates further overeating and eventual development of obesity and related EDs. Remarkably, the HPT is more responsive to caloric deficit than to caloric overload, which further poses a vulnerability factor in modern society, where highly processed and hyperpalatable food is easily accessible.^30^^,^^31^ However, apart from dysregulated eating behavior, cognitive, emotional, and psychological components also play a role in the development of ED. Indeed, individuals with EDs commonly show impaired inhibitory control, emotional dysregulation, and distorted self-perception, which facilitates the transition to an ED.^32-34^

### Bulimia nervosa

BN is defined by the loss of control over palatable food, which results in binge eating, characterized by recurrent episodes of excessive food consumption over a short period of time. Individuals with BN demonstrate an increased concern about their body weight or shape, which leads to guilt and shame after binge episodes and ultimately results in compensatory behaviors, such as self-induced vomiting or the use of laxatives, diuretics, and similar medications. Apart from the aforementioned behaviors, people with BN might also compensate for high calorie consumption by episodes of fasting or excessive exercise. Overall, such behaviors open the binge-purge cycle, facilitating the development of the ED.^5^^,^^35^ Based on the DSM-5, BN is confirmed by the presence of at least one binge-purge cycle per week for three months, while the frequency of the cycles defines the severity of BN.^36^ Furthermore, multiple self-report questionnaires have been developed for the successful diagnosis of BN and BED, including the Binge Eating Scale, the Eating Disorder Examination Questionnaire, and the Eating Loss of Control Scale, among others.^37^

Despite the consumption of high-calorie food, these individuals usually maintain stable body weight. However, BN is linked to multiple health outcomes, mainly affecting the cardiovascular and gastrointestinal systems, leading to electrolyte imbalance, metabolic and endocrine disturbances, as well as mouth and dental damage due to frequent contact with gastric acid.^38^^,^^39^ In addition, multiple brain areas are affected in BN patients, including the HPT, striatum, insula, amygdala, orbitofrontal and prefrontal cortices, among others. These neurobiological alterations are associated with the loss of control over food intake and the dysregulated reward and emotional processing observed in BN.^40-45^ Indeed, researchers observed that the risk of psychiatric disorders such as anxiety, depression, and emotional dysregulation is significantly increased in people with BN, raising the overall suicide rates compared to the healthy population.^38^

Currently, the gold standard for BN treatment is cognitive behavioral therapy. For pharmacological approaches, antidepressants are the first in line to decrease the number of binge episodes; thus, fluoxetine is currently the only FDA-approved drug for BN.^5^^,^^46^

### Binge eating disorder

In line with BN, BED is characterized by binge episodes occurring at least once per week for three or more months. The major difference from BN is that although individuals are distressed and often also experience feelings of shame and guilt related to binge episode, they do not indulge in compensatory behaviors; therefore, people with BED often have higher body weight and show an increased risk of developing obesity. For a positive diagnosis, in addition to the binge episode, individuals must show signs of loss of control, such as eating more rapidly, overeating until feeling uncomfortably full, eating when not hungry, or eating alone owing to embarrassment or guilt related to their eating behavior. The higher the number of binge episodes, the greater the severity of BED.^5^^,^^47^

Since obesity is a common comorbidity of BED, obesity-associated health outcomes are also linked to BED, including cardiovascular diseases, metabolic and hormonal alterations, as well as inflammatory stress, among others.^48^^,^^49^ Apart from peripheral alterations, changes in the CNS have also been described. Indeed, BED has been closely related to the dysregulation of the homeostatic and reward systems, as well as cortical impairments, all of which contribute to strong cravings, overconsumption of palatable food, and inability to stop once the eating process is initiated.^48^^,^^50-52^ Cognitive impairments further hinder treatment efficacy and contribute to relapse, as well as pose vulnerability risks toward the development of addictive behaviors. Ultimately, mood disorders are also commonly observed, with an increased prevalence of anxiety and depression, although suicide rates are lower compared to BN.^4^^,^^37^^,^^49^

Given the similar nature of BED and BN, cognitive behavioral therapy is also applied as a potential treatment strategy, especially if pharmacological treatment has not achieved a substantial effect. As for pharmacological strategies, numerous clinical trials have demonstrated a positive effect of topiramate and selective serotonin reuptake inhibitors. Other studies have investigated GLP-1R agonizts, which target the homeostatic system, as well as naltrexone/bupropion for the modulation of the reward system. However, currently, the only FDA-approved compound for BED treatment is lisdexamfetamine, which was proposed to act by increasing PFC activity and thus enhancing the inhibitory control.^5^^,^^50^

### Food addiction

FA is a type of maladaptive eating behavior characterized by the compulsive consumption of highly palatable food and the loss of control over its intake. Individuals with FA experience strong cravings for palatable food and related cues, impulsive consumption, withdrawal-like symptoms when food is unavailable, and eventual relapse.^21^^,^^53-56^ Despite sharing similar neurobiological mechanisms with drug addiction, FA is not yet included as an official ED in the DSM-5. Regardless, the DSM-5 criteria for substance use disorder have been adapted for FA, and the Yale Food Addiction Scale 2.0 (YFAS 2.0) was developed. YFAS 2.0 is currently the only available self-report questionnaire used to diagnose FA in humans.^9^^,^^57^

Long-term overconsumption of palatable foods in vulnerable individuals dysregulates the reward system, enhances incentive salience, and facilitates further overeating through positive reinforcement. Over time, these neuroadaptations contribute to a neurobiological shift from goal-directed to habitual behavior, as evidenced by increased persistence in palatable food intake. Persistent maladaptive changes in the reward system, together with a hyperactive stress system, especially when food is unavailable, ultimately induce negative emotional states, such as nervousness, anxiety, and irritability, which facilitate food consumption through negative reinforcement. Ultimately, impairment in executive control, such as loss of cognitive flexibility and top-down inhibitory control, facilitates compulsive consumption, cravings, and relapse.^28^^,^^58-60^ Preclinical findings further support these mechanisms, showing that chronic exposure to palatable food enhances anxiety-like states and reinstatement of food-seeking behavior, as evidenced by operant models assessing reward frustration, extinction, and relapse.^61-64^ Overall, FA is defined in a three-stage recurring cycle, where with every cycle the pleasurable feelings wane while the negative emotional state intensifies, ultimately leading to maladaptive changes in the brain, culminating in addiction.^65-68^

Health consequences related to FA overlap with those observed in other EDs.^69^ Similarly, given the behavioral and neurobiological overlap with obesity, BED, and addictions, treatment strategies focused on the aforementioned disorders could help to address FA, with the most widely suggested being the propagation of a healthy lifestyle, cognitive behavioral therapy, and neurofeedback training; however, to date, no FDA-approved treatment strategies to combat FA are available.^70-72^

## Distinction between the eating disorders

Due to overlapping mechanisms and health outcomes, it is important to clearly distinguish between all these EDs and FA for successful selection of the treatment approach (Table 1). First, the difference in prevalence among these EDs suggests their distinct nature. Indeed, across adult population studies, the lifetime prevalence was reported to be approximately 1%–3% for BN,^3^^,^^35^ 2%–4% for BED,^47^^,^^73^ and 9%–10% for FA.^7^^,^^8^

**Table 1.** Distinction between BN, BED, FA, and obesity.

Parameters	BN	BED	FA
Body weight	↔	↔, ↑	↔, ↑
Palatable food	+	+	+
Binge episodes	+	+	−
Compensatory behaviors	+	−	−
Body image disturbance	+	±	−
Guilt, shame	High	High/moderate	Moderate/low
Psychiatric comorbidities	+	+	+
Tolerance, withdrawal	−	−	+
Global prevalence	1%–3%	2%–4%	9%–10%

↔ stable, ↑ increased, + present, − absent, ± variable (inconsistent).

When compared separately, the major aspect that distinguishes BN from other EDs is that individuals with BN have a more fluctuating body weight, which usually remains within the healthy range owing to the indulgence in compensatory behaviors, which is uncommon in other EDs.^48^^,^^74^

Meanwhile, the strongest behavioral overlap is observed between BED and FA, since both disorders are of a compulsive nature, related to altered reward system responsivity to highly palatable foods. Scientists have demonstrated that 57%–68% of people who have FA also meet the criteria for BED.^75^^,^^76^ However, certain distinctions between these two EDs should be acknowledged. First, individuals with FA do not usually experience emotional aspects observed in people with BED, such as concerns about one's body weight or shape, guilt, and shame.^77^ The lack of emotional concerns in individuals with FA might reflect the lack of investigation rather than nonexistence, suggesting a necessity to further explore and expand the YFAS 2.0 diagnostic criteria. Second, although people with FA might consume higher amounts of food, they usually do not engage in binge episodes, further differentiating these two EDs. Lastly, individuals with FA might have tolerance or withdrawal symptoms, which are not common in people with BED.^78^

It has been observed that people with BED and FA frequently have higher body weight due to increased consumption of high-calorie, palatable food, thus demonstrating a strong overlap with obesity; however, obesity might be considered more as a consequence of an ED rather than an ED on its own.^12^ Having obesity as a comorbidity is linked to a lower quality of life and a higher co-occurrence of other mental health disorders.^48^ Epidemiological data support the distinction between obesity and EDs, demonstrating that 20% of individuals with FA also reached the threshold for obesity diagnosis.^79^ Conversely, 47% of adults with comorbid obesity were diagnosed as having FA, compared to 9.7% of healthy people, suggesting that obesity could be both a vulnerability factor and a consequence of FA.^7^ Interestingly, a recent study has found that among people with FA, 77% also had both obesity and BED, suggesting a strong overlap between these EDs and obesity.^80^

## Gut microbiota and underlying mechanisms

**Figure 2.**
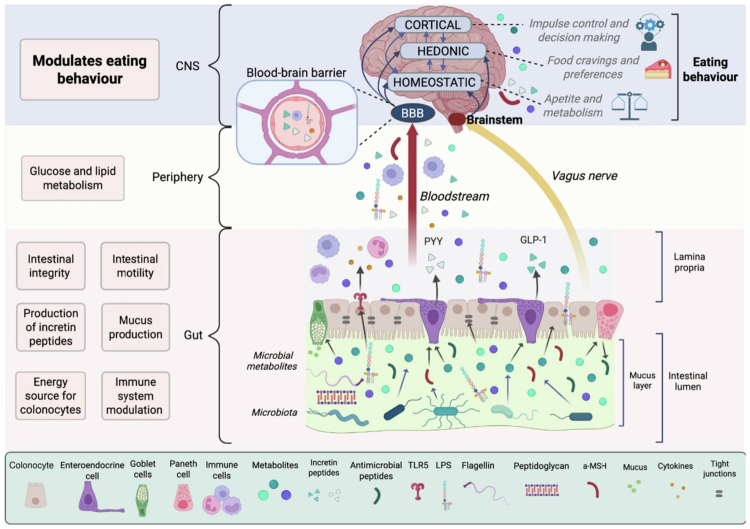
The role of the gut microbiota in eating behavior. The gut microbiota, localized in the intestinal lumen, produces various metabolites (blue arrows) that exert multiple functions in the gut (black arrows). Gut microbiota-derived molecules can alter brain function either directly, through the vagus nerve, which sends signals to the brainstem, or indirectly, by entering the blood circulation and crossing the BBB. Ultimately, microbiota-derived signals affect three major brain domains: homeostatic, responsible for appetite and metabolism, hedonic—food cravings and preferences, and the cortical control center, center for impulse control and decision making, thereby affecting overall eating behavior. On the left of the figure are depicted major gut microbiota-related functions in the gut, systemically, and in the CNS. PYY—peptide YY; GLP-1—glucagon-like peptide-1; TLR5—Toll-like Receptor 5; LPS—lipopolysaccharides.

Gut microbiota is a symbiotic community of bacteria, viruses, archaea, protozoa, and fungi, localized in the gut, with the highest abundance colonizing the cecum.^81^ By fermenting the non-digestible carbohydrates and other compounds, bacteria produce health-promoting metabolites such as short-chain fatty acids (SCFAs), neurotransmitters, their precursors, as well as vitamins, among others (Figure 2). SCFAs are a source of energy for colonocytes and are also involved in glucose and lipid metabolism. Apart from that, SCFAs and other metabolites interact with G-protein-coupled receptors localized on the apical side of enteroendocrine cells, resulting in the production of glucagon-like peptide-1 (GLP-1), peptide YY (PYY), and other satiety hormones and peptides, thus contributing to appetite regulation.^82-84^ Meanwhile, the microbiota-produced neurotransmitters, namely, GABA, dopamine, glutamate, and serotonin, stimulate the enteric nervous system and regulate gut motility, inflammatory response, nutrient absorption, and appetite hormone secretion. In the brain, these neurotransmitters may directly or indirectly modulate eating behavior.^85^^,^^86^ Although it is not clear whether these microbiota-derived neurotransmitters act in the CNS, their effects on the brain could be achieved through vagal signaling, immune system modulation, or the production of neurotransmitter precursors.^85^ Indeed, studies with germ-free (GF) mice have demonstrated that the absence of gut microbiota is associated with altered neurotransmitter receptor expression in the brain.^87^ Other microbiota-derived metabolites are involved in modulating the expression of tight junction proteins, thus regulating the epithelial integrity and permeability.^88^ Ultimately, the gut microbiota is crucial for intestinal immune system maturation and functioning, and protection against pathogens through the production of antimicrobial peptides, mucus, and competing for adhesion sites and nutrients with pathogenic bacteria.^89^^,^^90^

Studies confirm a bidirectional communication between the gut and the brain through endocrine, immune, and metabolic pathways, termed the microbiota-gut-brain axis.^91-94^ It has been shown that gut microbiota-derived metabolites and other components can communicate with the brain by stimulating the vagus nerve, which conveys the signal to the brainstem ^95^^,^^96^ or by entering systemic circulation, crossing the blood–brain barrier (BBB), and directly modulating brain functioning (Figure 2). For instance, SCFA acetate has been shown to directly act in the HPT,^97^ while another SCFA, butyrate, has been implicated in weight loss through the suppression of NPY orexigenic neurons in the arcuate nucleus of the HPT.^98^ SCFAs also play a role in the reward system, since the administration of SCFAs decreases the anticipation of palatable food reward in humans, along with decreased activity in the reward system.^99^

Interestingly, intestinal bacteria are also known to produce mimetic peptides, mimicking the function of alpha-melanocyte-stimulating hormone (α-MSH) and insulin, further contributing to homeostatic food intake regulation (Figure 2).^100-102^ Indeed, *Escherichia coli,* among other *Enterobacteriaceae* family members, has been shown to produce caseinolytic protease B homolog protein (ClpB). ClpB not only mimics the anorexigenic peptide α-MSH and produces central effects by inducing satiety but also acts peripherally by stimulating PYY production in the gut.^103^

Apart from bacterial metabolites, the microbiota can also transmit information through bacterial components, such as lipopolysaccharides (LPS), peptidoglycans, and flagellins (Figure 2).^104^ Remarkably, scientists have shown a direct communication mechanism between gut microbial components and the brain in the context of eating behavior. They concluded that bacterial flagellin, whose abundance increases with feeding, stimulates the pattern recognition receptor Toll-like Receptor 5 (TLR5), which is located on PYY-producing neuropod cells in the distal ileum and colon of mice. The activation of such cells results in PYY production, which consequently stimulates Neuropeptide Y receptor type 2 (Y2R) on the vagus nerve and sends a satiety signal to the brainstem. Behaviorally, this mechanism was linked to a decrease in food intake and meal size, with no overall effect on immune and metabolic responses.^105^ Another bacterial component, LPS, has been shown to induce obesity-like effects, namely, weight gain, increased intestinal and BBB permeability, insulin resistance, and inflammation,^106-108^ further confirming that the gut microbiota modulates both metabolic and feeding processes. A potential mechanism behind such an effect has been proposed by Huwart et al., who demonstrated that low-dose LPS diffusion activated the TLR4 pathway, mimicking the neuroinflammatory phenotype of obesity, and resulted in altered food-seeking behavior in mice.^109^

## Gut microbiota modulates eating behavior

Scientific evidence points to the gut microbiota as a crucial factor modulating brain functioning, while alterations in its composition might be directly associated with multiple brain disorders.^110-112^ These findings suggest that the gut microbiota can strongly affect eating behavior. Indeed, some bacteria can modulate our food choices by increasing cravings for the foods that promote their growth (Figure 2).^113^^,^^114^ For instance, scientists observed that only during the stationary phase of *E. coli* growth, which is achieved in response to nutrient availability, its surface proteins have been found to stimulate PYY and GLP-1 production, activating anorexigenic neurons in the HPT and suppressing appetite.^115^ A subsequent study with *Drosophila* spp. concluded that the microbiota is crucial for food preferences and choices. Scientists have found species-specific food preferences among *Drosophila* fruit flies. Specifically, *Drosophila sechellia* showed a preference toward an octanoid smell, which *D. melanogaster* considered aversive. However, after ten generations of fecal microbiota transplantation (FMT) from *D. sechellia*, *D. melanogaster* not only lose the aversiveness toward the octanoid acid smell but also develop a preference for it.^116^ A different group further explored microbiota-related food preferences in *Drosophila* by demonstrating that when lacking essential amino acids in their diet, flies increase their preference for protein-rich foods, a mechanism that was dependent on the gut microbiota.^117^

Studies using germ-free (GF) rodents are frequently used to confirm the crucial involvement of the gut microbiota in various peripheral and central health outcomes. For instance, evidence indicates that GF mice are inherently resistant to high-fat diet-induced obesity, thus usually displaying lower body weight and reduced white adipose tissue (WAT) mass, regardless of the diet consumed; meanwhile, FMT from conventionally raised mice rescues the phenotype.^118^ Scientists have further observed that GF mice contain a different lipid profile, with lower levels of triglyceride and cholesterol,^119^ and demonstrate impaired digestive absorption of nutrients, especially related to fats,^118^ which might explain the phenotype observed.

Apart from metabolic and homeostatic effects, GF mice also have alterations in the reward neurocircuitry.^120^ Indeed, an antibiotic cocktail, which is used to deplete the gut microbiota, led to a significantly higher consumption and motivation for highly palatable food. Such behavioral alterations were linked to higher activity in the mesolimbic system. Ultimately, the recovery of the gut microbiota composition by FMT restored healthy eating patterns.^121^ Similarly, antibiotic-induced gut microbiota depletion has been shown to increase palatable food intake in lean male mice ^122^ and in a BED mouse model.^123^

## Microbiota as a biomarker of disease

Given the multitude of factors involved in shaping the composition of the gut microbiota, the search for potential “healthy” and “pathological” gut microbiota composition has been challenging. Despite that, scientists define healthy microbiota composition as a diverse, abundant symbiotic community with a substantial absence of bacteria that are described to induce health disorders.^124^^,^^125^ For instance, a gut microbiota profile characterized by a high relative abundance of *Bacteroides* and a low proportion of *Faecalibacterium* has been linked to systemic inflammation,^126^^,^^127^ while a profile rich in polyketide synthase-positive *E. coli* and *Fusobacterium* spp. has been associated with colorectal cancer.^128^^,^^129^ On the other hand, beneficial bacteria that are known to exert health benefits have also been described, namely *Streptococcus thermophilus, Saccharomyces boulardii*, *E. coli* Nissle 1917, and several bacterial strains from the genera *Lactobacillus, Enterococcus, Bacillus,* and *Bifidobacterium*.^130^ A functional gut microbiota profile also helps to distinguish between health and disease states.^131^ Another important distinction of a healthy gut microbiota is its ability to resist stress-related compositional change, as observed by microbiota re-stabilization after antibiotic interventions.^132^

The Human Microbiome Project facilitated the advancement in the gut microbiota field by building the foundation for microbiota research and linking its composition with healthy and diseased states.^133^^,^^134^ Indeed, a growing body of evidence links the changes in gut microbiota composition with multiple diseases, suggesting that such alterations can be both a vulnerability factor as well as the consequence of disease development.^91^^,^^111^^,^^135^^,^^136^ Therefore, a distinction of gut microbiota profile between healthy and diseased conditions could further help to diagnose, prevent, and treat such disorders. However, the use of microbiota composition alterations alone as a biomarker of diseases remains challenging. Thus, the combination of multiple biomarkers, including microbial diversity, microbiota-derived metabolites, functional microbial profiles, and resilience measures, among others, may improve diagnostic and preventive prospects.

## Gut microbiota alterations in EDs

Diet is a crucial modulator of microbiota composition. Accordingly, compulsive EDs are closely linked to changes in the gut microbiota composition.^137^ The initial evidence, proving the involvement of the gut microbiota in regulating body weight and food intake, was derived from GF studies, which confirmed that GF mice tend to have lower body weights despite higher food consumption compared to conventionally raised mice. FMT from such mice resulted in a body weight increase in GF mice, similar to that observed in conventionally raised mice, together with a decrease in food intake. Such a contradiction between higher body weight despite a reduction in food intake was explained by a microbiota-related increase in energy harvest and energy storage.^138^ Apart from homeostatic processes, studies have also implicated the gut microbiota in cognitive performance.^139^^,^^140^ Indeed, individuals with obesity commonly have cognitive impairments, which have been associated with obesity-induced microbiota alterations, specifically through altered aromatic amino acid metabolism.^141^^,^^142^ Remarkably, FMT from obese adults with impaired memory performance or cognitive flexibility resulted in similar cognitive alterations in mice, confirming the crucial role of the gut microbiota in the development of cognitive impairments associated with obesity and EDs.^141^^,^^142^

Given the alarmingly high global prevalence of obesity, it is not surprising that most human and animal studies explore microbiota composition in individuals with obesity; meanwhile, other EDs are left underexplored. In 2020, a protocol for investigating the link between the gut microbiota and BN and BED disorders was published; however, the findings of such a study have not yet been reported.^143^ In the next section, we describe the existing knowledge behind microbiota alterations in EDs and FAs. Studies summarizing microbiota composition changes linked to EDs and FA in humans and *in vivo* models are depicted in Tables 2 and 3, respectively. It is important to pinpoint that it is correlational data, thus it does not allow the determination of whether the microbiota alterations observed are the consequences or causes of the disease.

### Bulimia nervosa

Restrictive eating patterns and compensatory behaviors such as vomiting or the use of laxatives have been associated with both gut microbiota dysbiosis and lower microbial diversity, which could potentially contribute to the severity of ED.^113^^,^^144^ Specifically, the bacterial peptide ClpB has been considered a crucial mediator of BN due to its mimetic abilities as well as its immunomodulatory effects. Indeed, ClpB can directly act on the HPT, mimicking α-MSH activity and modulating food intake and energy balance.^115^ Furthermore, the presence of ClpB has been directly connected with the formation of cross-reactive autoantibodies against α-MSH, owing to its similar epitope conformation to ClpB.^145^ Along with α-MSH-reactive antibodies, anti-ClpB IgG and IgM are also present in human plasma, although ED diagnosis was not found to affect their levels.^145^ Being the heat shock protein, ClpB increases under stressful conditions such as food restriction, gastrointestinal diseases, or antibiotic use; however, a greater increase has been observed in female rats, suggesting a sex-dependent effect. Indeed, females also contain higher amounts of ClpB- and α-MSH-reactive IgG as well as α-MSH-reactive IgM, while in males, only α-MSH-reactive IgG and IgM were found to be elevated.^146^ Such sex-dependent alterations observed may help to explain the higher prevalence of EDs among women. Furthermore, scientists have confirmed that while testosterone does not have any effect, estradiol significantly decreases ClpB protein but not mRNA levels in *E. coli* cultures.^146^

Remarkably, the structure and kinetics of ClpB differ among people with AN, BN, and obesity, showing the mechanistic distinction between obesity and other EDs. Indeed, individuals with obesity have lower levels of α-MSH cross-reactive IgG, with a lower dissociation rate and higher melanocortin-4 receptor activation threshold. This could explain impaired homeostatic food intake, altered energy expenditure regulation, and hyperphagia in adults with obesity. Conversely, increased melanocortin-4 receptor activation-induced satiety offers a plausible explanation for the periods of the dietary restriction phase of BN and AN.^147^

The other potential mechanism behind ClpB-induced α-MSH-reactive autoantibodies has been suggested by Fetissov & Hökfelt, who concluded that instead of inactivating α-MSH, antibodies can form an α-MSH/IgG complex, increasing α-MSH signaling and protecting it from plasma peptidases.^103^^,^^147^ Indeed, animal studies confirmed that the administration of *E. coli*, containing ClpB, induced α-MSH-reactive IgG and IgM autoantibodies and resulted in decreased meal size but an increase in meal number, with no overall effect on total food intake, while ClpB-deficient *E. coli* did not have such an effect. Remarkably, direct ClpB administration had the opposite effect, resulting in elevated levels of a-MSH-reactive IgG and increased meal size and overall food intake.^145^ Such inconsistency in the results suggests that feeding behavior might be altered depending on the type of autoantibodies formed.

Another potential mechanism is based on the fact that ClpB and α-MSH cross-reactive autoantibodies show a negative correlation with BN scores, suggesting their implication in the development of this ED.^148^^,^^149^ Researchers have hypothesized that, in some cases, autoantibodies might induce ClpB neutralization, which could be linked to a bulimic episode.^103^

To conclude, although multiple studies implicate ClpB and α-MSH cross-reactive autoantibodies in BN pathogenesis, the exact mechanisms by which they affect the BN phenotype are not yet fully understood. Current research on the microbiota signature underlying BN is also limited; however, existing human studies have focused primarily on female sex and adolescence, the major vulnerability factors for BN. For instance, one research team explored the microbiota profile of adolescent girls with BN and demonstrated a decrease in *Faecalibacterium*, *Bacteroides, Lachnospira, and Lachnospiraceae_UCG_001* and an increase in *Adlercreutzia, Dorea,* and *Blautia*.^150^ Similar to the results found in cases of obesity, *Fecalibacterium prausnitzii* was shown to be decreased, while *the Eubacterium hallii* group *(Anaerobutyricum hallii)* was increased in girls with BN.^150^ Such an alteration in the gut microbiota composition was associated with changes in tryptophan metabolism.^150^ Genome-wide association studies further confirmed BN-related changes in the gut microbiota; however, with limited overlap between other studies.^151^^,^^152^ Notably, contradictory results on the abundance of *Eubacterium_hallii_*group *(A. hallii*) were observed between the studies, which might point to the different ages of patients as the major determinant of the gut microbiota composition.^150^^,^^151^ A differential microbial profile was also observed in binge-purge AN, which is a form of AN that differs from BN only by a significantly lower body weight. Interestingly, a substantial increase in *Bifidobacterium* and a decrease in *Odoribacter* and *Haemophilus* were found.^153^

To date, no animal models to successfully recreate BN have been developed, thus limiting the search for microbial biomarkers to human studies only.

### Binge eating disorder

Given that human studies on BED are limited, no overlap in the gut microbiota composition has been observed yet, leaving the search for potential microbial biomarkers for future research.^144^^,^^154^^,^^155^ However, unlike for BN, animal models of BED are well developed, mainly implementing restriction-refeeding cycles with the incorporation of palatable foods. Animal studies with females have shown that, similar to obesity, BED is linked to changes in ß-diversity.^150^ Although two studies explored females only, the results behind α-diversity were contradictory.^150^^,^^156^ As expected, substantial changes in the gut microbiota composition were observed in mice fed an *ad libitum* cafeteria diet, when compared to controls. Interestingly, however, microbiota alterations were also induced by intermittent access to a cafeteria diet in comparison with the continuous availability of such food.^156^ Specifically, in a female study, intermittent access to palatable food increased *Porphyromonadaceae unclassified_*OTU35 and decreased *Coprobacter_*OTU66.^156^ Nevertheless, such changes have not been observed in a study in males, which did not find substantial changes between intermittent versus continuous access to palatable food, suggesting sex-dependent differences.^157^ Although alterations in the gut microbiota composition of mice with intermittent access to palatable food in comparison with standard chow have been linked to an increase in *Blautia, Collinsella,* and a decrease in *Escherichia/Shigella*.^157^ Apart from microbiota-related changes, peripheral and central alterations were also evaluated. For instance, in a study in females, *ad libitum* access to a cafeteria diet was associated with decreased short-term memory and altered glucose tolerance, while intermittent access did not alter memory performance or glucose tolerance in rats but had a stronger effect on increasing lipid profiles, as observed by elevated triglyceride levels, suggesting an eating pattern-related effect.^156^ Meanwhile, in a study in males, only metabolic changes have been found, namely an increase in body weight and elevated levels of the hormones leptin and insulin.^157^ Such differential health outcomes might be associated with the different gut microbiota profiles observed.

### Food addiction

A recent FMT study in mice has highlighted the involvement of the gut microbiota in excessive motivation for palatable food, suggesting that microbiota alterations might be one of the contributing factors to the development of FA.^158^ Indeed, changes in the gut microbiota composition have also been proposed as one of the contributing factors to the development of addictions.^159-161^ The introduction of YFAS 2.0-facilitated FA research in humans, in connection with gut microbiota research. Dong et al. have explored the microbiota composition among women with obesity, with or without FA diagnosis, and have found changes at the family, genus, and species levels, with *Bacteroides plebeius, Eubacterium biforme,* and *A. muciniphila* decreased, while *Alistipes massiliensis* increased in women with obesity and FA, further confirming the distinction between FA and obesity.^162^ Similarly, another study with women with FA showed a decrease in *A. muciniphila*, with no other overlapping alterations.^163^ However, apart from changes in microbiota composition, these women also had an increased lipid profile, altered glucose signaling, and elevated Bulimic and Emotional eating scores, suggesting an overlap between all EDs.^163^ A study by Castells-Nobau et al. has explored the population of both males and females with FA diagnosis and demonstrated bacterial changes at a family and genus level.^164^ However, in addition to bacterial alterations, they also demonstrated that certain viruses, namely *Microviridae, Gokushovirus* WZ-2015a, were increased in individuals with FA, being the first study so far investigating the virome in patients with EDs.^164^ Recent experimental work has further underscored the importance of the gut virome in microbiota-gut-brain communication, showing that alterations in viral populations can modulate behavioral, immune, and microbial responses to chronic stress.^165^ Ultimately, another human study explored the general population of both males and females with a FA diagnosis and have demonstrated certain microbial alterations, including a substantial decrease in the *Blautia* genus.^8^

Interestingly, similar observations were also made in mice, which scientists validated with the administration of *Blautia wexlerae* and potential prebiotics that increase *Blautia* sp. abundance in the gut. Remarkably, pro- and prebiotics successfully prevented the development of FA-like behavior in mice.^8^ However, in the aforementioned study, scientists only used male mice, and given the higher prevalence of FA among women, functional validation in female mice remains to be investigated. Lastly, one more study has demonstrated that *Parabacteroides* is positively correlated with palatable food preference in HFD-fed mice, suggesting its potential involvement in hedonic food intake.^122^ Despite a mouse model of FA-like behavior has been fully developed and described,^166^ no other studies so far have explored the link between the gut microbiota and FA-like behavior in an animal model.

## Microbiota-based interventions

**Figure 3.**
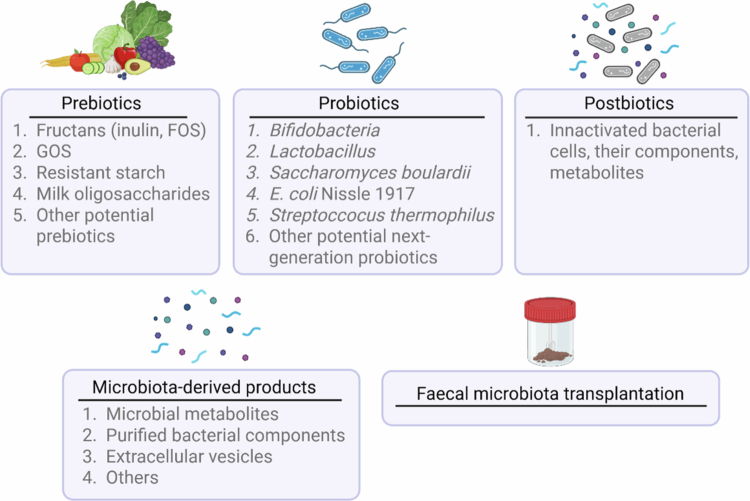
Microbiota-based interventions. Prebiotics, probiotics, postbiotics, microbiota-derived products, and FMT—the major microbiota-based strategies for modulation of the composition of the gut microbiota. Summarized examples of such microbiota-based interventions are described. FOS—fructooligosaccharides; GOS—galactooligosaccharides.

Multiple strategies have been developed over the years for effective modulation of the gut microbiota composition, achieving health benefits.^167^ Different microbiota-based interventions are summarized in Figure 3.

**Prebiotics** are defined as “a substrate that is selectively utilized by host microorganisms, conferring a health benefit.”^168^ Usually, prebiotics are water-soluble nondigestible compounds that are used as energy sources by microorganisms in the gut. The major group of prebiotics is fructans, which include inulin and fructooligosaccharides (FOSs). Other prebiotics are resistant starch, galactooligosaccharides (GOS), and human milk oligosaccharides, among others.^169-171^ Despite prebiotics being widely abundant in fruits and vegetables, owing to their relatively low concentrations and unhealthy human food preferences in a modern society, many prebiotics are currently available commercially as a food supplement.^171^^,^^172^

**Probiotics** are defined as “live microorganisms that, when administered in adequate amounts, confer a health benefit on the host.”^173^ Naturally, live microbial cultures are found in fermented products such as miso, kombucha, kimchi, yogurt, cheese, kefir, and sauerkraut, among others.^174-176^ However, because those naturally found microorganisms are not characterized at the strain level, they cannot be officially classified as probiotics. Apart from that, probiotics are widely available commercially. Specifically, *Lactobacillus, Bifidobacterium, S. boulardii*, *E. coli* Nissle 1917, *S. thermophilus,* and several other bacterial species and strains have been considered as probiotics and have been widely used as supplements.^172^

**Symbiotic** is a product of combining prebiotics with probiotics, which is believed to confer stronger health benefits. Positive effect is linked to increased survival and colonization of probiotics and the promotion of probiotic growth.^177^ Although existing pre- and probiotics have been shown to exert multiple benefits for the host, for better efficacy and succession rates, scientists are constantly searching for novel, next-generation probiotics and potential prebiotics.^178-180^

**Postbiotics** are defined as “a preparation of inanimate microorganisms and/or their components that confers a health benefit on the host.”^181^ Such suspensions can contain intact inactivated bacterial cells or their fragments, as well as their metabolites. However, the purified bacterial components or metabolites alone are not considered postbiotics by the International Scientific Association for Probiotics and Prebiotics, although the scientific community often refers to them as postbiotics.^181^^,^^182^ Postbiotics could be an efficient alternative to other microbiota-based interventions owing to their higher stability and easier transportation, storage, and standardization.^183^

Given that microbial metabolites, purified bacterial components, extracellular vesicles, and other products produced by intestinal bacteria do not fit into the postbiotic category, for the clarity and consistency of this review, we refer to them as microbiota-derived products.

**Fecal microbiota transplantation (FMT)** is a technique used to recolonize the gut microbiota by transplanting faces from healthy donors. Currently, the only available application of such a method is for the treatment of *Clostridium difficile* infection.^172^^,^^184^ A real-world example of potential applications of FMT for obesity and related EDs has been described by Alang&Kelly.^185^ Scientists have demonstrated that FMT alone can induce substantial changes in body weight since FMT from an overweight daughter to a mother infected with *Clostridium difficile* resulted in increased body weight of the mother.^185^ However, other studies did not corroborate the potential of FMT to transfer the obese phenotype,^186^ although FMT has been suggested to be successful in alleviating obesity and related metabolic disturbances.^187^

Given multiple risk concerns linked to FMT, especially the risk of transferring the microbiota from a donor with a predisposition to certain diseases, the FMT strategy is more widely investigated for the mechanistic perspective of gut microbiota and disease development, as well as to acknowledge its safety concerns and standardization.^188-191^

## Microbiota-based interventions in eating disorders

Given the alterations in gut microbiota composition observed in multiple human and animal studies of different EDs (Tables 2 and 3, respectively), this suggests that microbiota-based therapeutic approaches might have a beneficial effect in treating such disorders. The results of microbiota-based interventions in human and animal studies are depicted in Tables 4 and 5, respectively. Converging evidence from multiple studies suggests that the use of prebiotics, probiotics, symbiotics, postbiotics, microbiota-derived products, and FMT are successful strategies for reducing body weight and alleviating metabolic and behavioral outcomes related to EDs. Such a beneficial effect is often, but not necessarily, correlated with microbiota composition modifications related to the intervention used. Despite the considerable importance of such studies, certain limitations should be taken into account. First, the length of intervention strongly differs depending on the protocol used, which can vary from 2 weeks to 10 or more months, depending on the study. Such variability substantially determines the health consequences observed. Second, animal studies commonly use feces and cecum interchangeably for microbiota analysis, which induces strong variation in the microbiota results. Finally, there is a prevailing use of male rodents in *in vivo* studies, despite a substantially higher prevalence of these EDs being found in females. Such inconsistencies might contribute to the incoherence of the results and reduce the reliability; therefore, they should be taken with caution.

**Table 2.** Microbiota alterations in humans with eating disorders.

Disease	Study characteristics	Microbiota alterations	Other outcomes	Ref.
BN	✔ *N* = 20 ✔ ♀ ✔ Adolescents–young adults ✔ BN (DSM-IV)	Family: ↑ *Erysipelatoclostridiaceae* Genera: ↓ *Faecalibacterium*, *Bacteroides, Lachnospira, Lachnospiraceae_UCG_001* ↑ *Adlercreutzia, Dorea, Blautia* Species: ↓ *Fecalibacterium prausnitzii* ↑ *Eubacterium_hallii_group (Anaerobutyricum hallii)*	↓ Kynurenic acid	[149]
BN	✔ Genome-wide association studies (GWAS) datasets ✔ ♀, ♂ ✔ Adults, children ✔ BN	Genera: ↑ *Lonchococcus, Romboutsia* Species: ↓ *Eubacterium hallii (Anaerobutyricum hallii)*	N/A	[150]
BN	✔ Genome-wide association studies (GWAS) datasets ✔ ♀, ♂ ✔ Adults, children ✔ BN	Orders: ↓ Rhodospirillales ↑ Clostridiales Families: ↑ Ruminococcaceae ↓ Oxalobacteraceae, Lachnospiraceae Genera: ↑ *Coprobacter*, *Holdemania, Slackia*	N/A	[151]
Binge-purge AN	✔ *N* = 23 ✔ ♀ ✔ Adolescents, young adults ✔ BMI < 25 ✔ AN (DSM-5) ✔ AN vs AN + binge + purge	Orders: ↑ Bifidobacteriales ↓ Pasteurellales Families: ↑ Bifidobacteriaceae, Eubacteriaceae ↓ Pasteurellaceae Genera: ↑ *Bifidobacterium* *↓ Odoribacter, Haemophilus*	↑↓ Fecal metabolites	[152]
BN, BED	✔ *N* = 265 ✔ ♀, ♂ ✔ Adults ✔ BMI 30.4 ✔ BN/BED (DSM-5)	**Laxative use vs no laxative:** ↓ Alpha diversity Species: ↓ *Eubacterium ventriosum, Eubacterium alistipes, Bilophila*, *GCA900066575* **Vomiting vs no vomiting:** Genera: ↑*Escherichia-Shigella*	N/A	[192]
BN, BED	✔ *N* = 45 ✔ BMI 25.27 + -8.5 ✔ ♀ ✔ Adult ✔ Binge/purge vs healthy (DSM-5)	Family: ↑ Endomicrobiaceae Genus: ↑ *Prevotella*	↑ Depression ↑ Anxiety	[153]
BED	✔ *N* = 91 ✔ ♀, ♂ ✔ Adults ✔ Obese ✔ BED (DSM-5) ✔ Obese with BED vs obese without BED	Genera: ↓ *Sutterella, Akkermansia, Desulfovibrio, Intestinimonas* ↑ *Anaerostipes, Roseburia, Bilophila, Bifidobacterium*	= BMI ↓ Self-regulation score ↑ Reaction time in the inhibition task ↑ Mood disturbances	[154]
FA	✔ *N* = 105 ✔ ♀ ✔ Adults ✔ Obese ✔ YFAS (33.3%)	**Obese FA vs obese non-FA:** Family: ↑ *Ruminococcaceae* Genera: ↓ *Ruminococcus, Bacteroides, Desulfovibrio,* ↑ *Bacteroides*, *Megamonas, Odoribacter* *Species:* ↓ *Bacteroides plebeius, Eubacterium biforme, A. muciniphila* ↑ *Alistipes massiliensis*	↓ Indolepropionic acid	[193]
FA	✔ *N* = 100 ✔ ♀ ✔ Adults ✔ Lean, obese ✔ YFAS	Genera: ↓ *Ruminococcus, Coprococcus, Roseburia, Turicibacter, Adlercreutzia, Methanobrevibacter* Species: ↓ *A. muciniphila* ↑ *Ruminococcus torques*	↑ BMI ↑ Cholesterol ↑ TRIGL ↓ HDL ↑ Glucose ↑ Insulin ↑ YFAS 2.0 scores ↑ BITE score ↑ TFEQ emotional eating	[161]
FA	✔ *N* = 88 ✔ ♀, ♂ ✔ Adults ✔ Lean, obese ✔ YFAS 2.0	**Lean + obese + FA:** Phylum: ↑ Proteobacteria Species: ↓ *Blautia glucerasea, Lactobacillus kefiri*, *Amedibacillus dolichus* ↑ *Bordetella pertussis* **Obese + FA:** Genus/species: ↓ *Blautia* sp., *Blautia schinkii*, *Blautia wexlerae*	N/A	[8]
FA	✔ *N* = 88 ✔ ♀, ♂ ✔ Adults ✔ Lean, obese ✔ YFAS 2.0	Families: ↓ Aerococcaceae, Pasteurellaceae ↑ Planctomycetaceae Genus: ↑Anaerolineae bacterium Others (viruses): ↑ Microviridae*, Gokushovirus WZ-2015a*	N/A	[163]

BITE score—bulimic investigatory test Edinburgh; TEEQ score—three-factor eating questionnaire; DSM-IV—Diagnostic and Statistical Manual of Mental Disorders, Fourth Edition; DSM-5—Diagnostic and Statistical Manual of Mental Disorders, Fifth Edition; YFAS—Yale Food Addiction Scale; GWAS—genome-wide association study.

**Table 3.** Microbiota alterations in animal models of eating disorders.

Disease	Study characteristics	Microbiota alterations	Other outcomes	Ref.
BED	✔ *N* = 10 ✔ C57BL/6 ✔ **♀** ✔ Faces ✔ Binge protocol (diet + stress + palatable food)	↓ Alpha diversity Changes in beta diversity Families: ↓ Lactobacillaceae, Ruminococcaceae, ↑ Bacteroidaceae, Lachnospiraceae Genera: ↓ *Lactobacillus*, *Ruminococcaceae-UCG-014* ↑ *Bacteroides*, *Roseburia*, *Alistipes*	↑ Palatable food preference ↑ Cravings ↓ Kynurenic acid	[149]
BED	✔ *N* = 36 ✔ Sprague–Dawley rats ✔ **♀** ✔ Faces ✔ Groups: 1. Chow 2. Cafeteria diet (*ad libitum*) 3. Intermittent access to cafeteria diet	**Cafeteria vs control: (hedonic)** ↑ Alpha diversity Genera: ↓ *Alistipes* ↑ *Coprobacter*_OTU66, *Bacteroides* **Intermittent vs continuous: (binge)** Genera: ↑ *Porphyromonadaceae unclassified_*OTU35 ↓ *Coprobacter_*OTU66 **Intermittent and continuous:** Changes in beta diversity	**Cafeteria vs control:** ↓ Short-term spatial memory ↑ Body weight ↑ Glucose ↑ Astroglial and microglial proliferation genes in dHPC **Intermittent and continuous:** ↑ Fat mass ↑ Insulin ↑ Leptin ↑ TRIGL ↑ *dHPC proinflmmatory genes*	[155]
BED, FA	✔ *N* = 36 ✔ Sprague Dawley rats ✔ ♂ ✔ Faces ✔ Groups: 1. Chow 2. Cafeteria diet (*ad libitum*) 3. Intermittent access to cafeteria diet	**Cafeteria vs control: (hedonic)** Families: ↓ Enterobacteriaceae ↑ Coriobacteriaceae, Bacteroidaceae, Porphyromonadaceae Genera: ↓ *Escherichia/Shigella* ↑ *Blautia, Collinsella, Bacteroides, Ruminococcus* **Intermittent vs control: (binge)** Families: ↓ Ruminococcaceae, Enterobacteriaceae ↑ Coriobacteriaceae, Lachnospiraceae, Porphyromonadaceae Genera: ↓ *Escherichia/Shigella* *↑ Blautia, Collinsella*	**Cafeteria vs control:** ↑ Body weight ↑ WAT mass ↑ Leptin ↑ Insulin **Intermittent vs control:** ↑ Body weight ↑ WAT mass ↑ Leptin	[156]
FA	✔ *N* = 15 (recipients) ✔ FMT from HFD vs STD -fed donors ✔ C57BL/6 ✔ ♂ ✔ Cecum	Genus: ↑ *Parabacteroides*	↑ Palatable food preference	[122]
FA	✔ *N* = 24 ✔ C57 BL/6 ✔ ♂ ✔ Cecum ✔ Operant model of food addiction	Phylum: ↓ Actinobacteria Families: ↓ Coriobacteriaceae, Erysipelotrichaceae ↑ Anaeroplasmataceae Genera: ↓ *Lachnospiraceae UCG-001, Enterohabdus, Allobaculum, Blautia* *↑ Anaeroplasma*	N/A	[8]

WAT—white adipose tissue; dHPC—dorsal hippocampus; HFD—high-fat diet; STD—standard diet.

**Table 4.** Microbiota-based interventions in humans with eating disorders.

Disease	Study characteristics	Groups	Outcomes	Microbiota changes	Ref.
*Prebiotic interventions*					
Psychiatric aspects of EDs and FA	Randomized single-blind placebo-controlled trial. ✔ *N* = 106 ✔ Obese ✔ ♀, ♂ ✔ Adults ✔ Length of intervention—3 months	1. Placebo control group; 2. Prebiotic group (16 g/d inulin).	↓ Negative emotion ↑ Emotional competence ↑ Cognitive flexibility **In positive responders:** ↑ IL-8 ↑ DPP-IV ↓ WAT	Genera: ↑ *Bifidobacterium*, *Haemophilus*	[191]
FA	Randomized double-blind placebo-controlled trial. ✔ *N* = 59 ✔ Overweight ✔ ♀, ♂ ✔ Adults Length of intervention—2 weeks	1. Placebo control group; 2. Prebiotic group (30 g/d of inulin).	↓ Wanting scores ↓ Hunger ↓ Brain activity towards high caloric stimulus (VTA, OFC)	↓ alpha diversity Changes in beta diversity Phyla: ↓ Firmicutes ↑ Actinobacteria Family: ↑ *Bifidobacteriaceae*	[194]
Psychiatric aspects of EDs and FA	Open-label pilot study. ✔ *N* = 11 ✔ Overweight, obese ✔ ♀ ✔ Adults ✔ Length of intervention—1 month	1. Prebiotic group (glucomannan, oligofructose; SlimBiome®).	↓ Body weight^a^ ↓ Body mass^a^ ↑ Mood score^a^ ↓ Savory Craving Score^a^	Phylum: ↑Bacteroidetes, Actinobacteria^a^ Family: ↑Christensenellaceae^a^	[195]
*Probiotic interventions*					
BED	Randomized double-blind placebo-controlled trial. ✔ *N* = 152 ✔ Overweight ✔ ♀, ♂ ✔ Adults ✔ Length of intervention—3 months	1. Placebo control group; 2. Probiotic group *(*10 × 10^9^ CFU/d of *Lacticaseibacillus rhamnosus* HA-114).	= Body weight = Fat mass ↓ Insulin^a^ ↓ HOMA-IR^a^ ↓ LDL^a^ ↓ TRIGL^a^ ↓ Binge eating score^a^ ↓ Disinhibition score^a^ ↓ Food cravings^a^ ↓ Stress score^a^ ↓ Depression score^a^ ↑ Body esteem score^a^	↑ *Lacticaseibacillus rhamnosus*^a^^,^^b^ Microbiota analysis N/A	[196]
FA	Randomized double-blind placebo-controlled trial. ✔ *N* = 62 ✔ Obese ✔ ♀ ✔ Adults ✔ YFAS ✔ Length of intervention—3 months	1. Placebo control group; 2. The probiotic groups (1.8 × 10^9^ CFU/d) of *Lactobacillus acidophilus, Bifidobacterium bifidum*, *Bifidobacterium animalis* subsp. l*actis, Bifidobacterium longum*, *Lacticaseibacillus rhamnosus*, *Limosilactobacillus reuteri.*	↓ Body weight^b^ ↓ BMI^b^ ↑ Cognitive restriction score^a^^,^^b^ ↓ Hunger score^a^^,^^b^ ↓ Emotional eating score^a^^,^^b^ ↓ NPY^a^^,^^b^ ↓ Leptin^a^	N/A	[197]
FA	Randomized triple-blind placebo-controlled trial. ✔ *N* = 41 ✔ Weight regained after bariatric surgery ✔ ♀, ♂ ✔ Adults ✔ YFAS 2.0 ✔ Length of intervention—3 months	1. Placebo control group; 2. Probiotic group (1.8 × 10^9^ CFU/d of *Lactobacillus acidophilus, Bifidobacterium bifidum*, *Bifidobacterium animalis* subsp.*lactis, Bifidobacterium longum*, *Lacticaseibacillus rhamnosus*, *Limosilactobacillus reuteri).*	↓ Body weight^b^ ↓ BMI^b^ ↑ Cognitive restriction score^a^^,^^b^ ↓ Uncontrolled eating score^b^ ↓ Emotional eating score^a^ ↓ Appetite score^a^^,^^b^ ↓ Food addiction score^a^^,^^b^ ↓ Leptin^a^	N/A	[198]
BED, FA	Randomized double-blind placebo-controlled trial. ✔ *N* = 44 ✔ Obese ✔ ♀, ♂ ✔ Adults ✔ YFAS, BES ✔ Length of intervention—90 d	1. Placebo control group; 2. The probiotic groups (5 × 10⁹ CFU of *Lactobacillus acidophilus* NCFM, *Bifidobacterium animalis* subsp. *lactis* Bi-07).	↓ Food addiction score^a^^,^^b^ ↓ Binge eating score^a^^,^^b^	N/A	[199]
FMT interventions					
Psychiatric aspects of EDs and FA	Randomized double-blind placebo-controlled trial. ✔ *N* = 28 ✔ Obese, IR ✔ ♀, ♂ ✔ Adults ✔ Length of intervention—3 months	1. Control group (autologous FMT); 2. FMT group (FMT from healthy, lean donors).	**1 month:** ↓ LDL^a^ ↓ Kunurenine (faces)^b^ *↑* Indole acetic acid (faces)^b^ *↑* Butenylcarnitine (faces)^b^ **3 months:** ↓ Isoleucine (serum)^b^ ↓ Leucine (serum)^b^ ↓ Decenoylcarnitine (serum)^b^ ↓ Phenylacetic acid (faces)^b^ ↓ LDL^a^ ↓ Hunger^a^ ↓ Ghrelin^a^ ↓ Sweet craving^a^ *↑* Emotional well-being^a^ *↑* Energy level^a^ ↓ Anxiety (*p* = 0.056)^a^ ↓ Depression^a^	**1 month:** Genera: ↓ *Streptococcus*^b^ *↑ Coprococcus, Bifidobacterium, Bacteroides, Roseburia*^b^ Species: *↑ Bacteroides xylanisolvens, Lacto-coccus lactis*^b^ **3 months:** Genus/species: ↓ *Roseburia intestinalis*^b^ ↑ *Blautia obeum, Bacteroides*^b^	[200]

IL-8—interleukin-8 (pro-inflammatory cytokine); DPP-IV—dipeptidyl peptidase IV (glycemia and insulin resistance marker); WAT—white adipose tissue; HOMA-IR—homeostatic model assessment of insulin resistance (insulin resistance marker); TRIGL—triglycerides; VTA—ventral tegmental area; OFC—orbitofrontal cortex; LDL—low-density lipoprotein cholesterol; NPY—neuropeptide Y (orexigenic peptide); YFAS—Yale Food Addiction Scale; BES—Binge Eating Scale; IR—insulin resistance.

^a^ Compared to baseline (within-group).

^b^ Compared to placebo (between-groups).

**Table 5.** Microbiota-based interventions in animal models of eating disorders.

Disease	Study characteristics	Groups	Outcomes	Microbiota changes	Ref.
*Prebiotic interventions*					
Psychiatric aspects of EDs and FA	✔ *N* = 56 ✔ HFD-induced obesity ✔ C57BL/6 ✔ ♂ ✔ Cecum ✔ Intervention length—1-10 months	1. Obese control group; 2. Prebiotic group (3% of GOS, 3% of FOS).	**1 month:** no changes **10 months:** ↓ Anxiety-like behavior ↓ Oxidative stress (microglia) ↑ Short-term memory ↑ Spatial memory ↑ Physical activity ↑ Phagocytic efficiency (microglia) ↑ ccl2, cdkn2a, trem2, nox1 (microglia)	Changes in beta diversity Families: ↑ Muribaculaceae, Prevotellaceae, Rikenellaceae, Oscillospiraceae, Bifidobacteriaceae, Clostridiaceae, Eubacteriaceae, Lachnospiraceae, Bacteroidaceae ↓ Chlmydiceae, Peprostreptococcaceae	[201]
BED	✔ *N* = 12 ✔ Swiss Albino mice ✔ ♂ ✔ Cecum ✔ Intervention length—14 weeks ✔ 4 h of food deprivation + HFD	1. Vehicle control group; 2. Prebiotic group (10 mg/kg of cinnamaldehyde).	↓ Body weight gain ↓ Hyperphagia ↓ Leptin ↓ Fat mass ↓ IL-1β, MCP1, TNF-α, IL-6 (WAT) ↑ POMC, UCN, BDNF, CART, CCK (HPT)	No effect (*Lactobacillus, Bifidobacteria*, and *Roseburia* species altered by HFD were not affected by the intervention)	[202]
FA	✔ *N* = 24 ✔ C57BL/6J ✔ ♂ ✔ Cecum ✔ Intervention length—2 months *Ad libitum* palatable high-fat high-sugar diet.	1. Control group (high-fat high-sugar diet); 2. Prebiotic group (10% of FOS).	↓ NPY (HPT) **HFHS diet replaced with standard diet (10% of FOS):** ↓ Palatable food consumption and tropism ↑ DAT (*p* = 0.077), D1R (*p* = 0.0541) (NAc) ↑ NPY, AgRP (HPT)	Genera: ↑ *Bifidobacterium*, *Lactobacillus* Species: ↑ *A. muciniphila* **HFHS diet replaced with standard diet (10% of FOS):** Genera: ↑ *Bifidobacterium* Species: ↑ *A. muciniphila*	[203]
FA	✔ *N* = 41 ✔ C57BL/6J ✔ ♂ ✔ Faces ✔ Intervention length—120 d Operant model of food addiction	1. Control group; 2. Prebiotic (I) group (1% of rhamnose); 3. Prebiotic II group (1% of lactulose).	**Rhamnose:** ↓ Compulsivity-like behavior ↓ Food addictive-like behavior **Lactulose:** ↓ Food addictive-like behavior	**Rhamnose:** Genera: ↑ *Blautia*, *Selenomonadales, Faecalicatena, Parabacteroides* Species: ↑ *Blautia faecis*, *Parabacteroides goldsteinii, Alistipes dinegoldii* **Lactulose:** Genera: ↑ *Selenomonadales, Faecalicatena* Species: ↑ *Blautia pseudococcoides, Faecalibacterium prausnitzii*	[8]
*Probiotic interventions*					
Psychiatric aspects of EDs and FA	✔ *N* = 20 ✔ HFD-induced obesity ✔ C57BL/6 ✔ ♂ ✔ Intervention length - 8 weeks	1. Vehicle control group 2. Probiotic group (*Akkermansia muciniphila* MucT (2 × 10^8^ CFU/d)	↑ Motivation ↓ Tlr4, tlr2, CD45 (striatum) ↓ Ccl2 (striatum) ↓ Lpl (striatum)	N/A	[204]
BED	✔ *N* = 30 ✔ Wistar Kyoto rats ✔ ♂ ✔ Cecum ✔ Intervention length—21 d ✔ 12 h of food deprivation + 12 h of STD + 10% of sucrose	*1. Vehicle control group;* *2. Probiotic group (*1 × 10^8^ CFU/d of *Bacteroides uniformis* CECT 7771).	↓ Caloric intake (binge episode) ↓ Anxiety-like behavior = NPY, CART, AgRP, POMC ↑ DA, serotonin, noradrenaline (NAc) ↑ D1R (PFC)	Changes in beta diversity Family: ↑ Ruminococcacceae Genus: ↑ *Muribaculum* Species: ↑ *A. muciniphila, Christensenella minuta*, *Fecalimonas umblicata*	[205]
BED	✔ *N* = 45 ✔ C57BL/6J ✔ ♂ ✔ Faces ✔ Intervention length—2 weeks ✔ Antibiotic-induced binge eating	1. Vehicle control group (not treated with AB); 2. Control group (treated with AB); 3. The Probiotic I group (10^8^–10^9^ CFU of *A. muciniphila*, after AB treatment); 4. Probiotic II group (10^8^-10^9^ CFU of *Muribaculum intestinale* YL7, *Muribaculum intestinale* YL27, *Paramuribaculum intestinale* B1404, *Ligilactobacillus johnsonii,* after AB treatment).	**Probiotic I:** ↓ High-sucrose pellet consumption	**Probiotic II:** Genera/species: ↑ *Muribaculum intestinale* YL7, *Muribaculum intestinale* YL27, *Paramuribaculum intestinale* B1404, *Lactobacillus*	[120]
FA	✔ *N* = 16 ✔ C57BL/6J ✔ ♂ ✔ Intervention length—27 d ✔ 2% of sucrose *ad libitum*	1. Vehicle control group; 2. Probiotic group (1 × 10^10^ CFU of *Ligilactobacillus salivarius* LS7892/*Lactobacillus gasseri* LG6410).	= Body weight ↓ Sucrose intake before and during stress ↓ Depression-like behavior	N/A	[206]
FA	✔ *N* = 37 ✔ C57BL/6J ✔ ♂ ✔ Intervention length—120 d ✔ Operant model of food addiction	1. Vehicle control group; 2. Probiotic group (1 × 10^9^ CFU of *Blautia wexlerae*).	↓ Compulsivity-like behavior ↓ Motivation ↓ Food addiction-like behavior	N/A	[8]
*Postbiotic interventions*					
Psychiatric aspects of EDs and FA	✔ *N* = 35 ✔ HFD-induced obesity ✔ C57BL/6 ✔ ♂ ✔ Faces ✔ Intervention length—5 weeks	1. Vehicle control group; 2. The probiotic group (10^9^ CFUs/d of live *A. muciniphila);* *3.* Postbiotic group (10^9^ CFU/d of pasteurized *A. muciniphila);* *4.* Microbiota-derived products group *(*10 µg of *A. muciniphila* EVs).	**All experimental groups:** ↓ Body weight gain ↓ Glucose ↓ TRIGL, LDL ↓ TNF-α, IL-6 ↓ Intestinal inflammation ↓ Adipocyte size ↓ cldn-2, tlr-4, TNF-α, IL-10, angptl-4 ↑ IL-10 ↑ HDL ↑ zo-1, ocldn, cldn-1, tlr-2 **Postbiotics and microbiota-derived products:** ↓ Food intake ↓ Cholesterol	**Postbiotics and microbiota-derived products:** ↓ Firmicutes/Bacteroidetes ratio Phylum: ↓ Firmicutes Family: ↓ Prevotellaceae **Separate groups:** Phylum: ↑ Verrucomicrobiota (probiotics) Classes: ↓ Alphaproteobacteria (postbiotics) ↓ Gamaproteobacteria (probiotics and microbiota-derived products) ↓ Clostridia (microbiota-derived products) Genera: ↓ *Roseburia* spp. (probiotics, postbiotics) ↑ *Alistipes* spp. (probiotics) Species: ↓ *Escherichia coli*. (probiotics) ↑ *A. muciniphila* (probiotics)	[207]
Psychiatric aspects of EDs and FA	✔ HFD-induced obesity ✔ C57BL/6 ✔ ♂ ✔ Intervention length—30 d	1. Vehicle control group; 2. Probiotic group (1.5 × 10^9^ CFU/d of symbiotic probiotic communities).	↓ Body weight ↓ Body weight gain ↓ Cholesterol ↓ WAT ↓ Adipocyte size ↓ Anxiety-like behavior ↑ GLP-1	N/A	[208]
*FMT interventions*					
Psychiatric aspects of EDs and FA	✔ *N* = 9 ✔ HFD-induced obesity ✔ C57BL/6 ✔ ♂ ✔ Faces ✔ Intervention length—2 weeks	1. Obese control group; 2. FMT group (FMT from lean mice).	↓ Body weight gain ↓ Adipocyte size ↓ Astrogliosis (HPC) ↑ Glucose tolerance ↑ Long-term memory	Changes in beta diversity Phylum: ↑ Verrucomicrobiota ↓ Actinobacteria	[209]
Psychiatric aspects of EDs and FA	✔ *N* = 15 ✔ HFD-induced obesity ✔ C57BL/6 ✔ ♂ ✔ Cecum ✔ Intervention length—1 week	*1. Control group (FMT from STD-fed lean mice donors)* *2. Obese group (FMT from HFD-fed obese mice donors)*	= Body weight = Food intake ↓ Palatable food preference ↓ D1R, D2R, TH (striatum) ↑ DAT (striatum)	Genus: ↓ *Parabacteroides*	[122]

HDL—high-density lipoprotein cholesterol; TRIGL—triglycerides; LDL—low-density lipoprotein cholesterol; TNF-α—tumor necrosis factor-alpha (pro-inflammatory cytokine); CCL2—chemokine ligand 2 (monocyte chemoattractant); CDKN2A—cyclin-dependent kinase inhibitor 2 A (inflammatory/stress marker); TREM2—triggering receptor expressed on myeloid cells 2 (microglial/macrophage marker); NOX1—NADPH oxidase 1 (oxidative stress marker); WAT—white adipose tissue; GLP-1—glucagon-like peptide-1; ZO-1—zonula occludens-1 (tight junction protein); IL-1β—interleukin-1 beta (pro-inflammatory cytokine); IL-10—interleukin-10 (anti-inflammatory cytokine); MCP-1—monocyte chemoattractant protein-1 (monocytes/macrophages marker); POMC—pro-opiomelanocortin (satiety neuropeptide); UCN—urocortin (satiety/stress neuropeptide); BDNF—brain-derived neurotrophic factor (neuroplasticity and energy-balance marker); CART—cocaine- and amphetamine-regulated transcript (satiety neuropeptide); CCK—cholecystokinin (satiety hormone); HPT—hypothalamus; NPY—neuropeptide Y (orexigenic peptide); FOS—fructooligosaccharides; DAT—dopamine transporter (reward marker); D1R—dopamine D1 receptor (reward marker); D2R—dopamine D2 receptor (reward marker); AgRP—agouti-related peptide (hunger neuropeptide); NAc—nucleus accumbens; CLDN-2—claudin-2 (tight junction protein); TLR-4—toll-like receptor 4 (bacterial endotoxin sensor); TLR-2—toll-like receptor 4 (bacterial recognition receptor); ANGPTL-4—angiopoietin-like 4 (lipid metabolism and gut permeability regulator); OCLDN—occludin (tight junction protein); PFC—prefrontal cortex; HPC—hippocampus; HFD—high-fat diet; STD—standard diet, CD45 - cluster of differentiation 45 (immune cell marker); LPL—lipoprotein lipase extracellular enzyme).

### Prebiotics

The most widely investigated prebiotic for obesity treatment over the last 5 y is inulin.^194^^,^^210^ Given that obesity might be both a vulnerability factor as well as a consequence of EDs, inulin could potentially be used in alleviating symptoms related to EDs. Indeed, human studies have demonstrated that 3-month inulin supplementation improved cognitive flexibility and emotional competence, decreasing the negative emotional state in adults with obesity.^194^ Such behavioral outcomes could substantially improve weight loss by reducing the relapse rate, which is a common consequence of EDs.^211^ In a related study, only 2 weeks of inulin supplementation in overweight adults was sufficient to reduce hunger, wanting scores, and decrease brain activity in areas associated with reward, namely, the VTA and OFC, thus showing potential for application in FA. These behavioral outcomes were linked to modifications in ß-diversity, a decrease in α-diversity, and certain microbiota changes at the phylum and family levels.^195^ Interestingly, Visuthranukul et al. have performed a long inulin supplementation study for 6 months in children with obesity, and an increase in α-diversity, contrary to previous data, was observed, suggesting that the length of the intervention strongly determines the composition of the gut microbiota.^210^ However, the behavioral outcomes were not investigated in the present study. Apart from inulin, a prebiotic combination of glucomannan and oligofructose facilitated weight loss and induced changes in the gut microbiota composition along with an increase in the mood score and a decrease in the savory craving score, suggesting its potential applications for BED and FA.^212^

*In vivo* models have explored a wider range of prebiotic compounds and potential prebiotics. For instance, scientists have examined 14 weeks of supplementation with cinnamaldehyde as a potential prebiotic for treating BED. Although they observed a decrease in weight gain and hyperphagia, as well as a decrease in adipose tissue inflammation and an increase in satiety signaling in the HPT, no microbiota alterations induced by HFD were alleviated, raising the question of what mechanisms were behind its beneficial effect.^213^ In another study, the combination of FOS and GOS supplemented for 10 months improved short-term and spatial memory and reduced anxiety-like behavior—the frequent health outcomes related to EDs.^214^ The observed behavioral outcomes were linked to changes in beta diversity and microbiota composition at the family level.^214^

The prebiotic intervention for another ED, FA, has also been investigated *in vivo*. Indeed, FOS and the potential prebiotics lactulose and rhamnose have been explored. While, 2 months of supplementation with FOS increased *A. muciniphila*, no substantial behavioral or metabolic changes were observed until the highly palatable diet was replaced with standard chow.^215^ Meanwhile, 120 d of rhamnose and lactulose supplementation significantly prevented the development of FA-like behavior in an operant chocolate-flavor pellet self-administration paradigm. Interestingly, rhamnose also decreased compulsivity-like behavior, which represents a loss of inhibitory control and is closely linked to relapse in both addictions and EDs. Scientists have concluded that behavioral modifications are associated with changes in the gut microbiota composition, specifically an increase in *Blautia* genus abundance.^8^

### Probiotics

Multiple probiotics have been used in clinical trials for their therapeutic potential in EDs. For instance, *Lacticaseibacillus rhamnosus* HA-114 supplementation was used to treat BED.^198^ Although no changes in body weight or fat mass were observed after 3 months of intervention, substantial improvements in insulin tolerance and plasma lipids were found. Along with that, study participants showed a decrease in binge eating, disinhibition, stress, and depression scores, reduced food cravings, and increased body esteem, overall achieving successful improvement of the BED phenotype.^198^ Narmaki et al. investigated the combination of probiotics *Lactobacillus acidophilus, Bifidobacterium bifidum*, and *Bifidobacterium animalis* subsp. *Lactis, Bifidobacterium longum*, *L. rhamnosus*, *Limosilactobacillus reuteri* (formerly *Lactobacillus reuteri*) on FA in the context of obesity. They have demonstrated that 3 months of supplementation was successful in decreasing body weight and BMI, with a substantial effect on brain function. Indeed, women have shown improvements in cognitive restriction, a decrease in emotional eating, and hunger.^199^ Remarkably, these results were reproduced by Ghafouri-Taleghani et al., despite investigating both sexes, suggesting that probiotic-induced outcomes might have a stronger effect compared to sex-related changes.^204^ Finally, another human study incorporated obesity, FA, and BED patients and explored 90-d probiotic supplementation of *Lactobacillus acidophilus* NCFM and *B. animalis* subsp. *Lactis* Bi-07. The probiotic cocktail effectively decreased both FA and BED scores; however, metabolic and other parameters have not been investigated.^207^

A preclinical study reported that the administration of *A. muciniphila* to obese mice counteracted HFD-induced effects on reward processes, which were mediated through altered BBB permeability and inflammatory signaling in the striatum.^208^ Another *in vivo* study investigated the combination of symbiotic probiotic communities with HFD-induced obesity, administered for 30 d. They have demonstrated improved weight loss, decreased body weight gain and lipid profile, as well as elevated satiety hormone GLP-1 levels. Additionally, a reduction in anxiety-like behavior was observed.^205^ Consequently, other animal studies have further explored the potential of probiotic supplementation for EDs. A recent preclinical study on BED investigated probiotics, namely, *Bacteroides uniformis* CECT 7771,^216^ *A. muciniphila*, ^121^ and the cocktail of *Muribaculum intestinale* YL7, *M. intestinale* YL27, *Paramuribaculum intestinale* B1404, and *Ligilactobacillus johnsonii,* on BED-related outcomes.^121^ In the first study, 21 d of *Bacteroides uniformis* CECT 7771 supplementation was linked to a lower calorie intake during a binge episode, lower anxiety-like behavior, and an increase in DA, serotonin, and noradrenaline signaling in the NAc, together with upregulation of D1R in the PFC. The observed effects were associated with changes in microbial ß-diversity and an increase in *A. muciniphila, Christensenella minuta*, and *Fecalimonas umblicata* in the rat cecum.^216^ Given that antibiotic use induces binge eating behavior in mice, scientists have used this model to explore whether probiotic administration of *A. muciniphila* or a cocktail of *M. intestinale* YL7, *M. intestinale* YL27, *P. intestinale* B1404, and *L. johnsonii* alleviates antibiotic-induced BED. Scientists have concluded that only 2 weeks of *A. muciniphila* administration significantly reduced high sucrose-containing pellet consumption compared to both the healthy control and the antibiotic-induced binge-eating control group. However, the cocktail of *M. intestinale* YL7, *M. intestinale* YL27, *P. intestinale* B1404, and *L. johnsonii* sustained a stronger effect on microbiota recovery after antibiotic-induced depletion, with a significant increase in *M. intestinale* YL7, *M. intestinale* YL27, *P. intestinale* B1404, and *Lactobacillus*.^121^

Regarding FA, 27-d administration of *Ligilactobacillus salivarius* LS7892/*Lactobacillus gasseri* LG6410 to sugar craving mice significantly decreased sugar intake before and during a stressor, and overall decreased depression-like behavior.^217^ Lastly, *Blautia wexlerae* was used as a potential probiotic in a mouse model of FA-like behavior, and 120 d of supplementation resulted in a decrease in compulsivity-like behavior and motivation towards chocolate-flavor pellets, and overall decreased food addiction-like behavior, suggesting its potential therapeutic applications for FA management.^8^

### Postbiotics and microbiota-derived products

Postbiotics, such as inactivated bacteria or their lysates, as well as microbiota-derived products such as bacterial metabolites, extracellular vesicles, and other molecules, have been investigated as potential therapeutic strategies for counteracting EDs. Nevertheless, both human and animal studies exploring the potential of postbiotics, or microbiota-derived products, are scarce.

Interestingly, it was observed that postbiotics might carry stronger therapeutic potential compared to probiotics, since in a human trial, 3 months of supplementation with pasteurized *A. muciniphila* had a stronger effect than live *A. muciniphila* in reducing lipid and glucose levels, improving insulin resistance, and decreasing inflammation, with no changes in microbiota composition.^218^ However, the results were not reproduced in an *in vivo* model, since all experimental groups demonstrated a similar effect.^206^ In addition, the administration of pasteurized *A. muciniphila* and its extracellular vesicles reduced food intake in mice, demonstrating a stronger effect on homeostatic food intake regulation.^206^ Given that inactivated bacteria might exert a stronger beneficial effect, it should be more extensively explored in the context of EDs. However, to date, no studies focusing on the use of postbiotics for treating EDs have been described.

### FMT

A limited number of FMT studies have explored the microbiota transplantation as a potential therapeutic strategy for EDs. Scientists have described a human study in which they performed autologous FMT for the control group with obesity, while the experimental group received FMT from healthy, lean donors.^219^ One month after FMT, changes in microbial metabolites in human faces were observed, namely, a decrease in kynurenine and an increase in indole acetic acid and butenylcarnitine, together with a decrease in *Streptococcus* and an increase in *Coprococcus, Bifidobacterium, Bacteroides,* and *Roseburia.* Interestingly, a more substantial effect was found after 3 months, with a decrease in serum isoleucine, leucine, and decenoylcarnitine. Behavioral modifications after 3 months of intervention were also described. Namely, a decrease in hunger, together with lower levels of ghrelin, lower sweet craving, improved emotional well-being, higher energy levels, and a decrease in anxiety and depression scores. Remarkably, the improvement was linked to a decrease in *Roseburia intestinalis* and an increase in *Blautia obeum* and *Bacteroides*.^219^

Studies on FMT interventions in HFD-induced obese mice have demonstrated a reduction in body weight and adipocyte size, along with an improvement in glucose tolerance and long-term memory comorbidities of the EDs, suggesting the potential of FMT in alleviating health consequences related to EDs.^220^ Another study has implemented the FMT approach and observed that palatable food preference positively correlated with *Parabacteroides* abundance, suggesting the potential involvement of this bacterium in hedonic food intake.^122^

## Conclusions and future directions

Scientific evidence confirms that the gut microbiota plays a major role in homeostatic, reward, and executive processes associated with EDs and FA. Both human and *in vivo* correlational studies confirmed a link between EDs and gut microbiota alterations, which might point to both causal and consequential relationships (Table 6). To date, ClpB remains the strongest predictor for BN development. However, a decreased abundance of *Fecalibacterium* might be another potential biomarker of this disease. While biomarkers related to BED are still lacking, a lower abundance of *Blautia* and *Akkermansia* has been suggested as a potential indicator of FA.

**Table 6.** Summarized findings on gut microbiota alterations and microbiota-based interventions for different EDs.

EDs	Microbiota alterations	Microbiota-based interventions *(in humans)*		Microbiota-based interventions *(in animal models)*		
		Prebiotics	Probiotics	Prebiotics	Probiotics	Others
BN	↑ ClpB ↓ *Fecalibacterium*	Glucomannan + oligofructose; inulin	–	–	–	Pasteurized *A. muciniphila* and EVs of *A. muciniphila*, symbiotic probiotic communities, FMT interventions
BED	(?)		*L. rhamnosus* HA-114; *L. acidophilus NCFM, B. animalis subsp. lactis Bi-07*	Cinnamaldehyde	*B. uniformis* CECT 7771; *A.muciniphila*	
FA	↓ *Blautia* ↓ *Akkermansia*		*Bifidobacterium* and *Lactobacillus* strains*, L. rhamnosus, L. reuteri; L. acidophilus* NCFM, *B. animalis* subsp. *lactis* Bi-07	Rhamnose; lactulose	*L. salivarius* LS7892 + *L. gasseri* LG6410; *B. wexlerae*	

Ultimately, microbiota-based therapeutic strategies have proven to be a safe and effective adjuvant therapies for officially approved treatments (Table 6). The most promising strategy is prebiotic inulin and the next-generation probiotic *A. muciniphila,* which might produce an even stronger effect when pasteurized, opening the opportunities for postbiotic-based therapeutic interventions that prove to be safer and cost-effective alternatives to current probiotics. However, further research is needed before it can be successfully translated to clinical and commercial applications.

The existing scientific data highlight certain drawbacks of these studies, mainly related to the lack of standardization in the intervention length and fecal or cecal use for microbiota analysis.

Furthermore, the variability in response to microbiota-targeted intervention limits the potential of translational applications of such approaches. Another concern is the predominance of studies conducted exclusively on male animals, which introduces bias and limits the translational value, especially given that EDs show higher prevalence in women. Moreover, the available scientific data raise questions about translatability between human and animal studies, which might be linked to the aforementioned limitations. Lastly, despite abundant data in the context of obesity, the clinical studies on BN, BED, and FA are still lacking, establishing directions for future research.

Overall, despite existing limitations, these studies contribute to scientific progress towards the search for potential microbiota-based biomarkers for EDs and therapeutic interventions for disease prevention and treatment.

## Abbreviations

AN: anorexia nervosa
BED: binge eating disorder
BBB: blood–brain barrier
BN: bulimia nervosa
ClpB: caseinolytic protease B homolog protein
DSM-5: diagnostic and statistical manual of mental disorders—5th edition
ED: eating disorder
FA: food addiction
FMT: fecal microbiota transplantation
FOS: fructooligosaccharides
GF: germ-free
GLP-1: glucagon-like peptide-1
GLP-1R: glucagon-like peptide-1 receptor
GOS: galactooligosaccharides
HFD: high-fat diet
HPT: hypothalamus
YFAS 2.0: Yale food addiction scale 2.0
LPS: lipopolysaccharides
SCFA: short-chain fatty acid
PYY: peptide YY
WAT: white adipose tissue
α-MSH: alpha-melanocyte-stimulating hormone
