# Correction

**DOI:** 10.1080/19490976.2026.2667667

**Published:** 2026-05-05

**Article title:** Dysregulated eating behaviour and microbiota-based interventions targeting eating disorders and food addiction

**Authors**: Samulėnaitė, S., Rodriguez Parkitna, J., Baltriukienė, D., Martín-García, E., Maldonado, R., & Burokas, A.

**Journal**: *Gut Microbes*

**Bibliometrics:** Volume 18, Number 01, pages 1—47

**DOI:** http://doi.org/10.1080/19490976.2026.2647535

This article was first published with errors in Tables.

The correct tables with citation numbering have been updated.

The article has been republished with errors corrected.

